# CircKIF4A enhances osteosarcoma proliferation and metastasis by sponging MiR-515-5p and upregulating SLC7A11

**DOI:** 10.1007/s11033-022-07296-2

**Published:** 2022-05-17

**Authors:** Pan He, Feng Liu, Zhijun Wang, Haoli Gong, Meilan Zhang, Zhen Jia, Xiaohui Zhai

**Affiliations:** 1 Department of Orthopedics, Hunan Provincial People’s Hospital, The First-Affiliated Hospital of Hunan Normal University, Changsha, 410005 China; 2 Cancer Research Institute, Hengyang Medical School of University of South China, Hengyang, China, Hengyang, China; 3 Department of Medical Oncology, The Sixth Affiliated Hospital of Sun-Yat Sen University, Guangzhou, 510655 China

**Keywords:** circKIF4A, Circular RNAs, SLC7A11, Competing endogenous RNAs, Osteosarcoma

## Abstract

**Background:**

Circular RNAs (circRNAs) are forms of non-coding RNAs that have crucial roles in regulation of various biological processes of several malignant tumors. circKIF4A is closely associated with malignant progression of a variety of cancers. However, the molecular mechanisms as well as roles of circKIF4A in osteosarcoma (OS) have not yet been clearly elucidated.

**Methods:**

We evaluated the expression of circKIF4A in OS. Colony-formation, cell counting kit-8 (CCK-8), transwell and mice metastasis model assays were done to explore the roles of circKIF4A in vitro and in vivo. TargetScan database, double luciferase, quantitative reverse transcription polymerase chain reaction analysis (RT-qPCR), and RNA immunoprecipitation (RIP) were done to investigate the associated molecular mechanisms.

**Results:**

In both OS cells and tissues, circKIF4A (hsa_circ_0007255) was found to be upregulated. In vitro and in vivo, circKIF4A knockdown markedly suppressed OS proliferation as well as metastasis. circKIF4A enhanced OS growth as well as metastasis by sponging miR-515-5p and by upregulating SLC7A11.

**Conclusions:**

We identified the biological significance of the circKIF4A-miR-515-5p-SLC7A11 axis in OS cell proliferation and metastasis, which is important in OS monitoring and treatment. More studies on circKIF4A will inform on the diagnostic markers for early OS screening.

**Graphical abstract:**

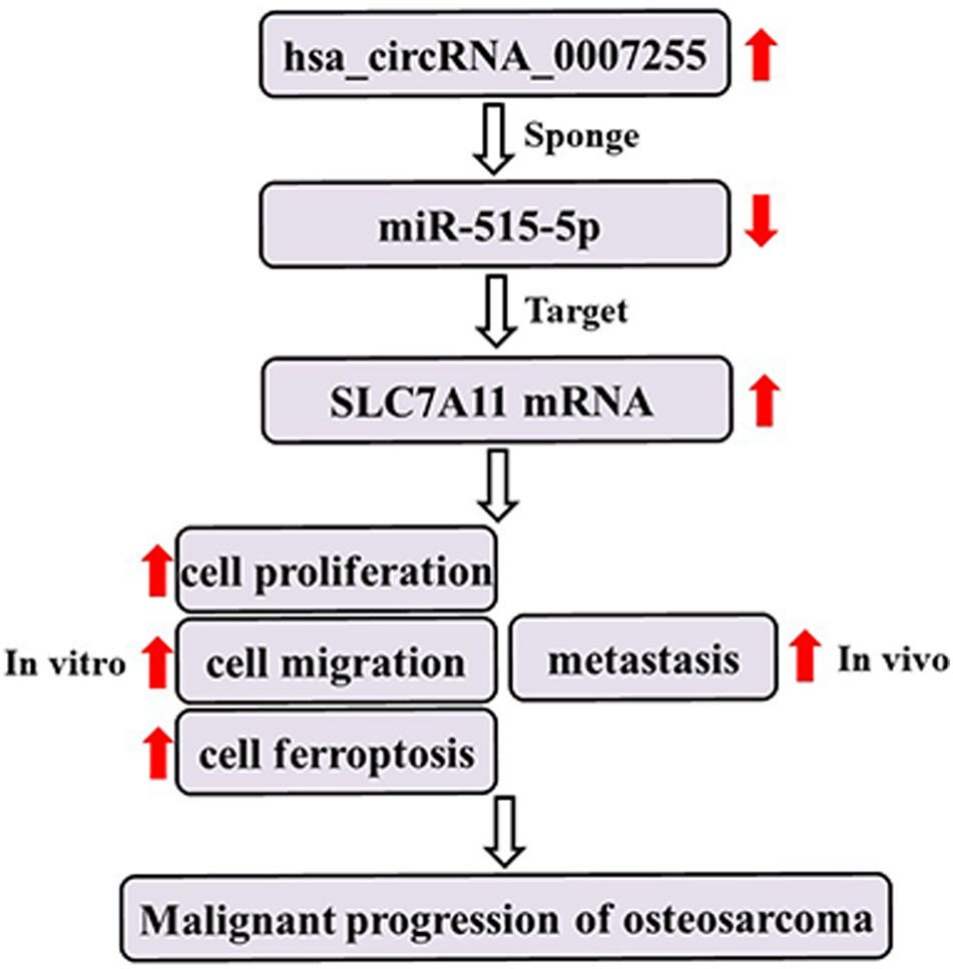

## Introduction

Osteosarcoma (OS), a primary malignant bone tumor whose origin is in the mesenchymal tissues, occurs more often in the long bones of children and young people under 25 [1]. The cause and mechanism of its pathogenesis are not yet clear. At present, the accepted treatment mode for osteosarcoma is surgical resection combined with preoperative and postoperative neoadjuvant chemotherapy. Although the chemotherapy regimen continues to improve and the treatment effect is improved, the 5-year survival rate for patients with osteosarcoma is still 60–70% [2]. Mainly due to the side effects of chemotherapeutics and the lack of specific molecular targets. Therefore, it is important to find specific molecular targets for osteosarcoma treatment, and then to develop targeted drugs with exact curative effect and high safety, which will improve the prognosis and survival rate of patients with osteosarcoma.

Circular RNAs (circRNAs) are abundant non-coding covalently closed RNAs. They are composed of exon or intron precursor mRNA reverse splicing sequence, without upstream head or downstream tail [3]. Compared with the linear mRNA, their structure, which is circular, enhances their stability and resistance to RNA exonucleases [4]. As novel forms of endogenous non-coding RNAs, their expressions in various cells and tissues are elevated, and they are potential miRNA sponges for regulating the expression of several key downstream genes, splicing or acting as transcription factors encoding proteins [5]. Due to advances in circRNA-sequencing as well as bioinformatics analyses, more and more abnormally expressed circRNAs in tumors have been identified [6]. CircRNAs are involved in many biological processes of malignant tumors [7].

circKIF4A (hsa_circRNA_0007255, chr13: 69549254-69553539) is derived from the exon region of the KIF4A gene. In glioma tissues, circKIF4A levels are significantly increased. Inhibition of circKIF4A can suppress clone formation, migration as well as invasive abilities of glioma cells, and increase their apoptosis [8]. In ovarian cancer tissues, circKIF4A levels are elevated. CircKIF4A knockdown suppresses ovarian cancer cell proliferation as well as migration, and as a kind of ceRNA, circKIF4A can upregulate JAM3 levels via sponge adsorption of miR-127 to enhance ovarian cancer progression [9]. In addition, circKIF4A can upregulate NOTCH2 levels by sponge adsorption of miR-375/1231 to enhance bladder cancer progression [10]. In triple-negative breast cancer, the circKIF4A/miR-375/KIF4A axis can regulate its progress through competitive endogenous RNA, and circKIF4A is a novel prognostic marker as well as treatment target for TNBC [11]. In addition, circKIF4A silencing can regulate ZEB1 expression through sponge adsorption of miR-152 to hinder cell metastasis and promote cell apoptosis in breast cancer [12]. circKIF4A promotes malignant progression of papillary thyroid cancer by sponging miR-1231, GPX4 upregulation and suppresses ferroptosis [13]. However, the functions and molecular mechanisms of circKIF4A in bone growth and metastasis of OS are still unclear. Based on the above research background, we evaluated the significance of circKIF4A on OS growth as well as metastasis, and explored the possible mechanisms of circKIF4A in regulating the progression of OS.

## Materials and methods

### Tissue samples

Thirty pairs of fresh OS tissues with their matching non-cancerous bone tissues were acquired from the department of orthopedics in Hunan Provincial People’s Hospital. This study was performed in accordance with the Declaration of Helsinki (as revised in 2013) and was permitted by the Ethical Committee of Hunan Provincial People’s Hospital. Informed consents were obtained from all patients prior to their inclusion. Study protocols were prepared prior to the study without registration.

### Cell culture

OS cell lines SOSP-9607, HOS, U2OS, SW1353, and Saos-2, as well as the normal human osteoblast line hFOB1.19 were bought from the American Type Culture Collection (ATCC, Manassas, VA, USA). Cell line maintenance and culture was done as instructed by ATCC. Cell authenticity was confirmed by DNA fingerprinting.

### RT-qPCR

The TRIzol reagent (Sigma, USA) was used for total cellular RNA extraction. The SYBR Premix Ex Taq Kit (Takara, Japan) was used for RT-qPCR assays. The primers used in this study were: SLC7A11, F: 5′-*GGTGGTGTGTTTGCTGTC*-3′, R: 5′-*GCTGGTAGAGGAGTGTGC*-3′ and GAPDH, F: 5′-GACCCCTTCATTGACCT-3′, R: 5′-CCACCACCCTGTTGCTGT-3′. The circRNA quantification process was comparable to that of mRNA. Before transcription, treatment with RNase R was done to remove linear RNA. Gene expressions were calculated using the 2^−ΔΔCt^ method and GAPDH was used as the mRNA and circRNA control while U6 was the miRNA control.

### Subcellular location of circRNA

Treatment with RNase R was used to assess circKIF4A stability. First, for 20 min, total RNA (1 µg) was treated with RNase R (3U/µg, Epicentre Technologies, USA) at 37 °C. Then, purification was done using the the RNeasy MinElute Cleanup Kit (74204, Qiagen, Germany), as instructed by the manufacturer. After transcription, expression levels of circKIF4A as well as its linear gene, KIF4A, were evaluated by RT-qPCR.

### Western blot analysis

Extraction of cellular proteins was done using the RIPA lysis buffer and the phenylmethylsulphonyls fluoride (PMSF) reagent. Separation of the target protein was done using sodium dodecyl sulfate polyacrylamide gel electrophoresis (SDS-PAGE) gel and polyvinylidene fluoride (PVDF) membranes. Transfer conditions were 200 mA for 2 h on PVDF membranes. Overnight incubation of the membranes was done in the presence of anti-SLC7A11 and anti-β-actin antibodies (1:2000) at 4 °C, after which they were incubated with specific secondary antibodies at room temperature (RT) for 3 h.

### RNase R digestion assay

Total RNA (5 µg) from OS cells Saos-2 and U2OS were treated with the control solution or RNase R (8.5 U/µg) and incubated for 30 min at 37 °C. The resultant RNA solution was purified, followed by RT-qPCR quantification.

### Actinomycin D assay

Linear mRNA transcription in OS cells U2OS and Saos-2 were digested by exposure to 5 ug/mL of actinomycin D for 0 h, 8 h, 16 h, or 24 h. Then, Saos-2 and U2OS cell lines were harvested at set time points. Quantification of linear KIF4A mRNA as well as the circular circKIF4A was done by RT-qPCR.

### Cell counting kit-8 assay

The U2OS as well as Saos-2 OS cell lines were digested and resuspended. Then, seeding of si-circCTR and si-circKIF4A transfected cancer cells in a 96-well plate (10^3^ cells/well) was followed by incubation at 37 °C. Prior to measurements, 10 µL of the cell counting Kit-8 (CCK-8) solution was added into every well, followed by 1 h of incubation at 37 °C [14, 15].

### Clone formation experiment

Logarithmic growth phase U2OS and Saos-2 cells were digested using 0.25% trypsin, beat them into individual cells and dilute it in gradient multiples for 3 parallel samples each group. The cells of each group were seeded in a six-well plate (100 cells per well), gently shake the cells to spread them evenly. Then place them in a 5% CO2 environment at 37 °C, and incubated until there were macroscopically visible clones in the cell culture plate. Subsequent stain the clones using crystal violet, after which colony counting was microscopically performed.

### Quantitative analysis of GSH/GSSG

Operate based on the manufacturer’s instructions (ab138881, Abcam, USA). Add the deproteinized samples and standards into wells. Thiol Green was added in the assay buffer for GSH detection, or add GSSG probe for total glutathione (GSH + GSSG) detection. Incubate the mixture at RT for 30 min. Then analyze the samples using a microplate reader. Calculation of GSSG levels was done by subtracting GSH from the total glutathione levels.

### Transwell assay

The OS cells (10^4^ cells/well) were resuspended after which they were transferred into upper chambers filled with serum-free medium. Fetal bovine serum (FBS) medium (20%) was loaded into the lower chambers. Then, cell fixation was done in methanol and stained with crystal violet (0.5%). Cell imaging and counting was done by microscopy [16] (Olympus IX73, Japan).

### Dual luciferase reporter assay

The U2OS as well as Saos-2 OS cell lines were seeded in 96-well plates (10^3^ cells/well). The constructed reporting vectors (circKIF4A-wt/mut or SLC7A117 3′-UTR-wt/mut), the miRNA mimics, as well as their controls were transfected into OS cells by Lipofectamine 3000. Cell harvesting was done after 48 h for the assessment of Renilla and firefly luciferase activities via the Dual-Luciferase Reporter Assay System (E1910, Promega, USA). Determination of relative luciferase activities was done by normalization of the firefly luciferase activities to Renilla luciferase activities.

### RNA immunoprecipitation (RIP)

The RIP assays were conducted using the Anti-Ago2 antibody. Expression levels of circKIF4A, SLC7A11 and miR-515-5p were determined after RNA purifications. Transfection of the MS2bs-circKIF4A vector, MS2bs-circKIF4A-mt vector as well as the MS2bs-Rluc vector into U2OS and Saos-2 cells was followed by 48 h of incubated. Then, the RIP assay was conducted. miR-515-5p abundance was evaluated after purification of the RNA complexes.

### RNA pull-down assay

In this assay, U2OS as well as Saos-2 (10^4^ cells/well) were first transfected with the biotin-labeled miR-515-5p or the miR-negative control (RiboBio, China). Cells were harvested after 48 h, lysed using the lysis buffer followed by 2 h of incubation in the presence of streptavidin beads (Thermo Scientific Fisher, USA) at RT. Then, beads were obtained and washed twice using PBS. Finally, the RNeasy Mini Kit (74,104, Qiagen, Germany) was used to clean eluted RNAs that had been attached to the beads. Finally, the abundance of SLC7A11 was assessed by RT-qPCR.

### Mouse metastasis model

Take 6-week-old nude mice and randomly divide them into 2 groups with 5 mice in each group. The U2OS OS cells (2 × 10^6^ cells/well) stably inhibiting circKIF4A (sh-circKIF4A) as well as its control (sh-CTR) were injected into nude mice with 200 µL of cell suspension through the tail vein [17]. After 4 weeks the mice were sacrificed. There were 5 pairs of formalin-fixed paraffin-embedded specimens, after deparaffinization, hydration and blocking were performed. HE stained lung metastasis specimens from the experimental group were compared with the control specimens under the microscope. The stained lung specimens were used for microscopic counting of metastatic sites.

### Statistical analysis

Data were analyzed using SPSS 23.0 and shown as mean ± SD. Comparisons of means between groups was done by the student’s *t*-test. Statistical significance was set at P ≤ 0.05.

## Results

### CircKIF4A is upregulated in OS

**Fig. 1:**
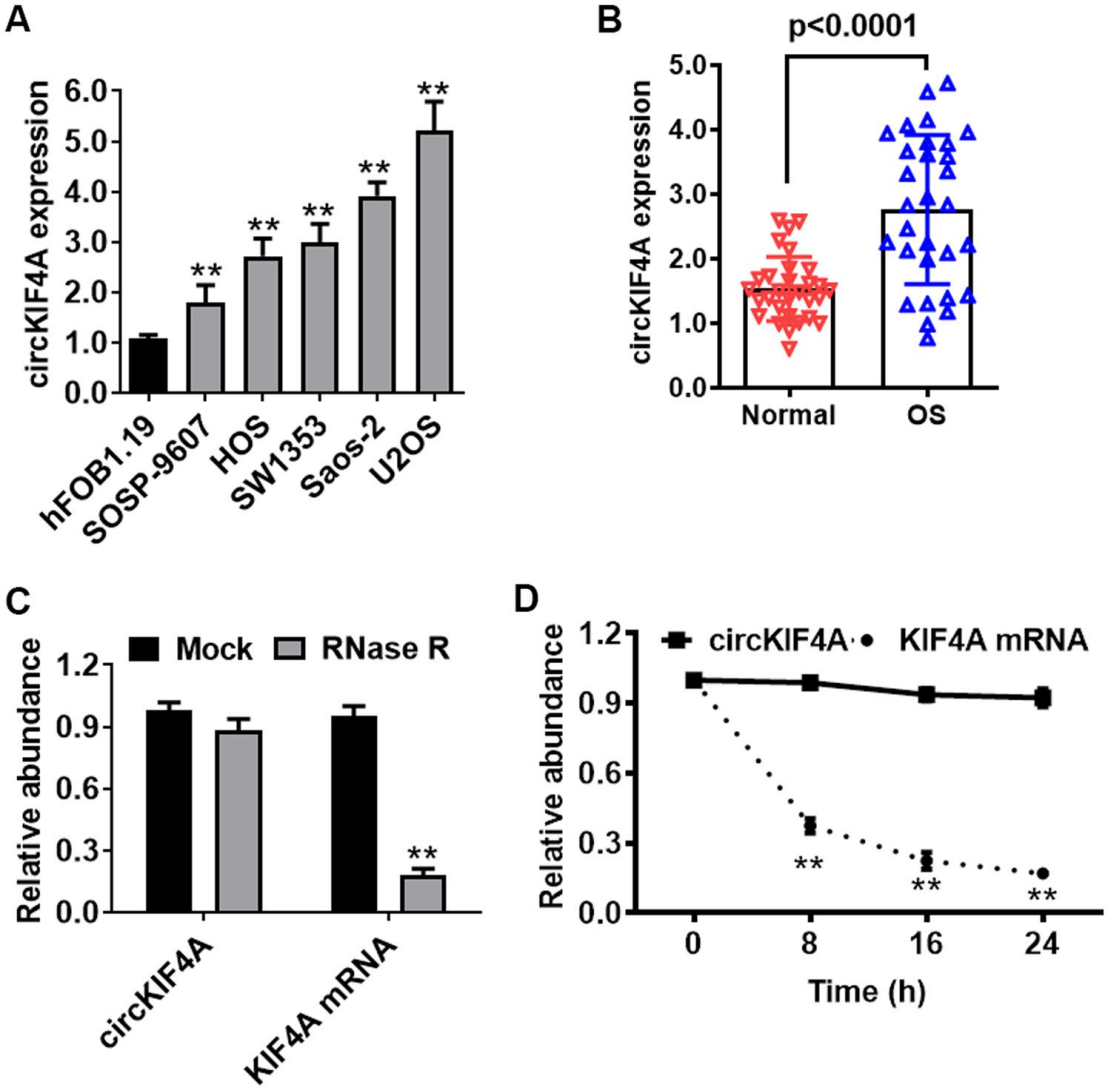
CircKIF4A is elevated in OS. **A** CircKIF4A levels were significantly upregulated in SOSP-9607, HOS, SW1353, Saos-2 and U2OS cells by qPCR. **B** circKIF4A was significantly upregulated in OS patient samples by qPCR. **C** The circular nature of circKIF4A was assessed by RNase R assay in Saos-2. **D** Circular transcripts of circKIF4A were found to be more stable than the linear KIF4A mRNA transcripts in Saos-2 cells by actinomycin D treated assay. **P < 0.01

We explored circKIF4A levels in OS cells and osteoblasts. Relative to normal human osteoblast hFOB1.19, circKIF4A was markedly upregulated in SOSP-9607, HOS, SW1353, Saos-2 as well as U2OS cell lines, particularly in U2OS and Saos-2 cells, detected by RT-qPCR (Fig. 1A). Thirty pairs of OS patient samples and corresponding non-cancerous bone tissue samples were collected and used for expression analysis of circKIF4A. circKIF4A was markedly elevated in all OS tissues (Fig. 1B). The circular structure as well as stabilities of circKIF4A were assessed by actinomycin D and RNase R assays. circKIF4A exhibited a high resistance to RNA exonuclease (RNase R) (Fig. 1C). Consistently, the circular form of circKIF4A was associated with a longer half-life span, relative to linear KIF4A mRNA (Fig. 1D).

### CircKIF4A silencing inhibited OS cell growth and metastasis

**Fig. 2:**
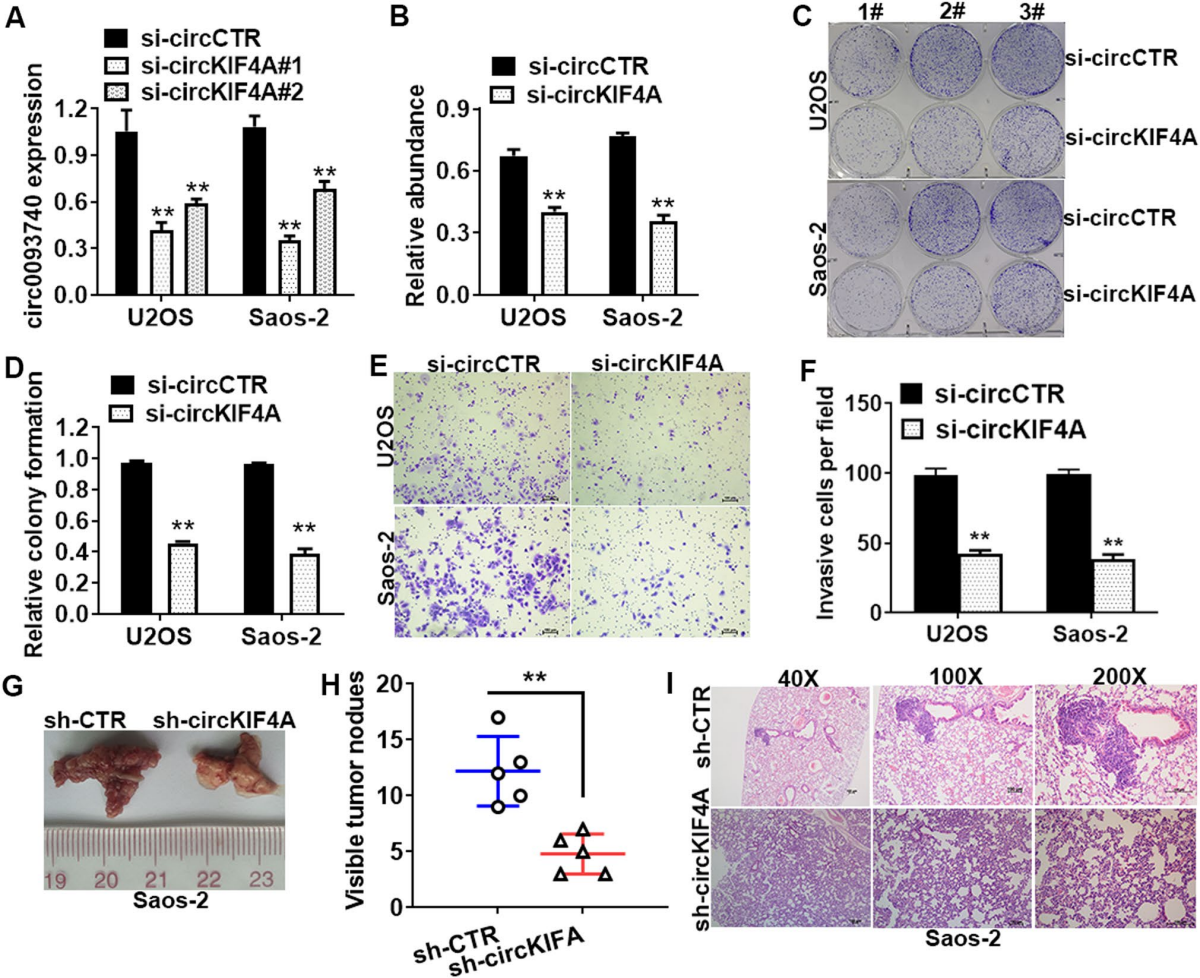
CircKIF4A knockdown suppressed OS cell growth and metastasis of OS cells. **A** Efficacies of si-circKIF4As were verified in U2OS as well as Saos-2 cells by RT-qPCR. **B** CCK-8 assay was conducted to assess U2OS and Saos-2 cell proliferative abilities. **C** The pictures of clones formed from OS cells was shown. **D** Statistical chart of Colony-formation assay was conducted to investigate the proliferation abilities of Saos-2 and U2OS cells. **E** The pictures of invasion cells from U2OS and Saos-2 cells was shown. **F** Statistical chart of Transwell assay was perform to investigate the migration abilities of Saos-2 and U2OS cells. **G** The representative pictures of lung tissues are shown. **H** Lung metastases were counted and documented. **I** Images of the stained sections of lung metastases [hematoxylin and eosin (HE) staining]. **P < 0.01

To evaluate the roles of circKIF4A in OS progression, circKIF4A was knocked down in U2OS as well as Saos-2 cells using the small interfering RNA (siRNA) targeting circKIF4A (si-circ_0007255) (Fig. 2A). The CCK-8 assays revealed that circKIF4A silencing markedly suppressed U2OS and Saos-2 cell proliferations (Fig. 2B). The colony-formation assay confirmed these results (Fig. 2C, D). To determine if circKIF4A silencing inhibited OS cell invasion, invasion assays were conducted. circKIF4A silencing suppressed U2OS and Saos-2 cell invasions in vitro (Fig. 2E, F). In addition, circKIF4A silencing inhibited the metastatic abilities of the U2OS cell line (Fig. 2G). The results demonstrated that lung metastasis nodule counts were remarkably low in the sh-circKIF4A group, relative to the sh-CTR group (Fig. 2H). HE staining was used to show the morphology of lung tissues and the number of nodules (Fig. 2I).

### CircKIF4A is a sponge of miR-515-5p in OS

**Fig. 3:**
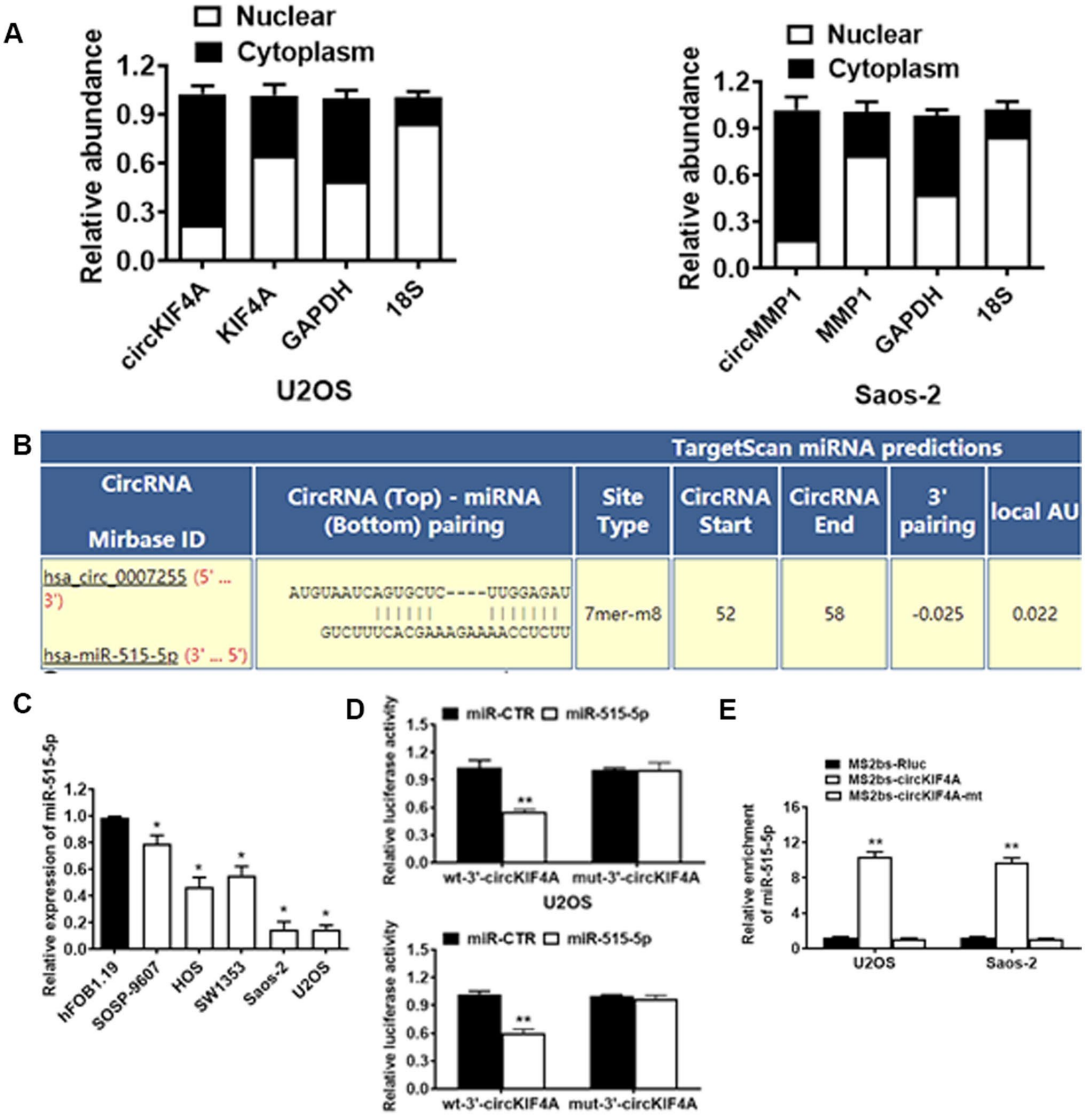
CircKIF4A is a sponge for miR-515-5p in OS. **A** 18 S, GAPDH, circKIF4A and KIF4A levels in cytoplasmic and nuclear fractions were evaluated in U2OS and Saos-2 cells by RT-qPCR. **B** Predicted binding sites for miR-515-5p in the circKIF4A sequence. **C** miR-515-5p levels in OS cells SOSP-9607, HOS, SW1353, Saos-2 and U2OS were significantly decreased compared to normal human osteoblast hFOB1.19. **D** Luciferase reporter assay of U2OS as well as Saos-2 cells co-transfected with the miR-515-5p mimics and the 3′-circKIF4A wild type or the mutant luciferase reporter. **E** MS2-based RIP assay transfected with MS2bs-circKIF4A, MS2bs-circKIF4A-mt or Rluc control plasmids. *P < 0.05, **P < 0.01

circKIF4A was shown to be localized in the cytoplasms of U2OS and Saos-2 cell lines (Fig. 3A). Potential interactions between multiple miRNAs and circRNAs were assessed using the Circular RNA Interactome database. Among the miRNAs, miR-515-5p bound the circKIF4A sequence with a potential interacting site (Fig. 3B). Relative to the normal human osteoblast hFOB1.19, miR-515-5p was all significantly suppressed in SOSP-9607, HOS, SW1353, Saos-2 as well as U2OS cell lines (Fig. 3C). Then, we determined if miR-515-5p could bind to circKIF4A. The relative luciferase activity was markedly suppressed upon co-transfection of miR-515-5p with the WT 3′-circKIF4A plasmid, when compared to transfection with the MUT 3′-circKIF4A plasmid (Fig. 3D). To confirm direct interactions between circKIF4A and miR-515-5p, RIP assay to pulldown circKIF4A was performed. It was found that miR-515-5p was highly upregulated in the MS2bs-circKIF4A overexpressed group in OS cells U2OS and Saos-2 (Fig. 3E).

### CircKIF4A enhances OS progression via the circKIF4A-miR-515-5p-SLC7A11 axis

**Fig. 4:**
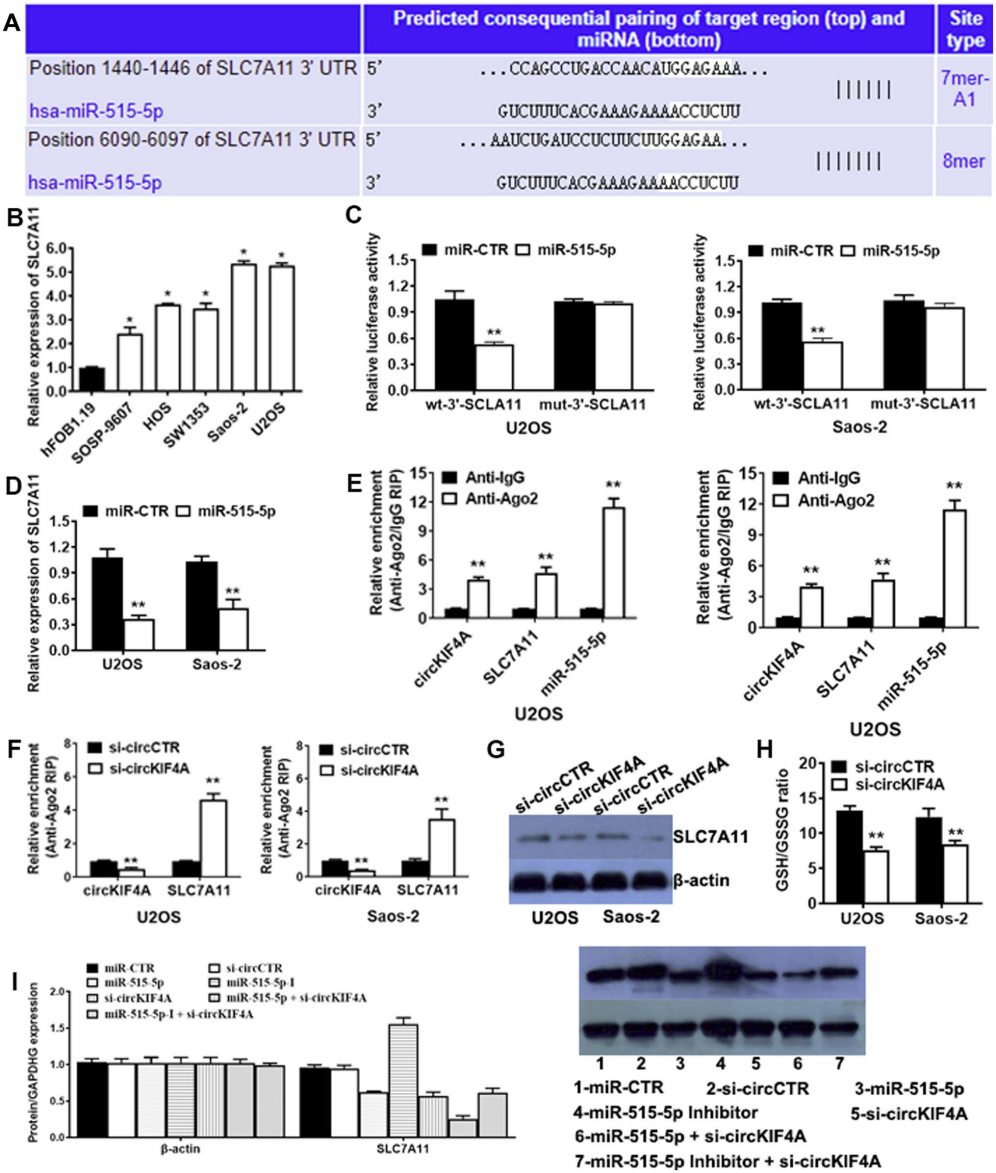
CircKIF4A enhances OS progression via the circKIF4A-miR-515-5p-SLC7A11 axis. **A** Based on the TargetScan website, SLC7A11 was predicted as the downstream target for miR-515-5p. **B** SLC7A11 levels in OS cells SOSP-9607, HOS, SW1353, Saos-2 and U2OS were significantly increased relative to those of hFOB1.19. **C** Luciferase reporter assay of U2OS as well as Saos-2 cells co-transfected with miR-515-5p and 3′-UTR of SLC7A11 mutant/wild type luciferase reporter. **D** Overexpressed miR-515-5p suppressed SLC7A11 Saos-2 and U2OS cells by RT-qPCR. **E** Enrichment of circKIF4A, SLC7A11 as well as miR-515-5p on AGO2 as evaluated by the RIP assay. **F** Enrichment of SLC7A11 to AGO2 was enhanced upon circKIF4A knock-down. **G** circKIF4A knockdown suppressed SLC7A11 protein levels in U2OS as well as Saos-2 cells. **H** circKIF4A knockdown decreased the GSH/GSSG ratio in Saos-2 and U2OS cells. **I** SLC7A11 protein levels were detected by western blot analysis in miR-CTR, si-circCTR, miR-515-5p, miR-515-5p inhibitor, miR-515-5p + si-circKIF4A and miR-515-5p inhibitor + si-circKIF4A groups. *P < 0.05, **P < 0.01

Potential targeting genes for miR-515-5p were determined by TargetScan (http://www.targetscan.org), microT (https://bio.tools/DIANA-microT), miRDB (http://mirdb.org) and miRMap (https://mirmap.ezlab.org) databases. The data indicated that SLC7A11 contains conserved binding sites for miR-515-5p in the intersection of the results of these four databases (Fig. 4A). Compared with hFOB1.19, SLC7A11 was all significantly upregulated in SOSP-9607, HOS, SW1353, Saos-2 and U2OS cells (Fig. 4B). Then, we determined if miR-515-5p could directly bind the 3′-UTR of SLC7A11 mRNA. The relative luciferase activity was markedly suppressed upon cotransfection of miR-515-5p with the WT 3′-UTR-SLC7A11 plasmid, relative to the MUT 3′-UTR-SLC7A114 plasmid (Fig. 4C). Meanwhile, compared with the miR-CTR group, exogenous miR-515-5p contributed to suppression of SLC7A11 mRNA expression levels (Fig. 4D). The RIP assay revealed that circKIF4A, SLC7A11 as well as miR-515-5p were highly enriched in the anti-AGO2 group in Saos-2 and U2OS OS cells (Fig. 4E). Enrichment to RNA induced silencing complex (RISC) of SLC7A11 was significantly elevated after circKIF4A silencing whether it’s in Saos-2 and U2OS cells (Fig. 4F). After transfecting with si-circKIF4A in OS cells, SLC7A11 protein levels were markedly decreased. That’s to say, circKIF4A silencing markedly suppressed SLC7A11 protein levels (Fig. 4G). Moreover, circKIF4A knock-down suppressed total GSH/GSSG ratios in Saos-2 and U2OS cells (Fig. 4H). Exogenous miR-515-5p inhibitor (HmiR-AN0571-SN-10, the control was CmiR-AN0001-SN, GeneCopoeia, China) rescued the expression of SLC7A11 which is reduced by si-circKIF4A, while the expression of SLC7A11 was the lowest when miR-515-5p and si-circKIF4A were co-introduced at the same time, comparing with the miR-CTR, si-circCTR, miR-515-5p, miR-515-5p inhibitor and si-circKIF4A groups (Fig. 4I).

## Discussion

OS, which is the most prevalent primary solid malignant tumor of bone tissues, is also one of the main causes of death in bone tumor patients [18, 19]. OS has a high degree of malignancy and early metastasis. It is easy to relapse and metastasize after treatment. The patient’s prognostic outcomes for OS are poor and the 5-year survival rate is relatively low. Invasion as well as metastasis mechanisms of OS are yet to be fully established, and it is still a hot and difficult point of current research [20, 21]. Thus, it is important to identify specific molecular targets for OS treatment, and then to develop targeted drugs with exact curative effect and high safety, which will improve the prognostic outcomes as well as survival rates for OS patients.

CircRNAs are highly expressed in cells and tissues, and are potential miRNA sponges for regulating the expressions of various key genes closely relatively to malignant tumor [22]. circKIF4A is significant in malignant progression of a variety of tumors [8–13]. We tested circKIF4A levels in OS cells and tissues. circKIF4A was markedly upregulated in OS. Multiple studies have shown that the expression of circKIF4A was up-regulated in various tumors, it was relatively cancerous [8–13]. Subsequently, siRNA and shRNA were used for circKIF4A knockdown in OS cells, the results revealed that circKIF4A silencing remarkably reduced the effect of circKIF4A on the growth, clone formation, invasion and lung metastasis in vitro as well as in vivo. Thus, we investigated its molecular mechanisms. CircRNAs regulate and communicate among themselves via competitive binding to shared miRNAs according to the ceRNA theory [23, 24]. We found that circKIF4A is an miR-515-5p sponge in OS, and regulatory relationships were verified via the dual luciferase reporter as well as RIP assays. Relative to circKIF4A, miR-515-5p expressions were markedly decreased in OS, further verifying the combination of the two. miR-515-5p inhibits various cancer processes, for instance, it acts as a prostate cancer inhibitor by targeting TRIP13 [25], suppresses HCC migration as well as invasion via the IL6/JAK/STAT3 pathway [26], inhibits breast cancer cell proliferation, migration as well as invasion via the hsa_circ_0008039/miR-515-5p/CBX4 axis [27]. The functions of miR-515-5p in OS is basically the same as the above researches.

We established that miR-515-5p binds SLC7A11 3′-UTRs, and its expression was confirmed to be markedly suppressed by miR-515-5p. SLC7A11 is a molecule closely related to ferroptosis, a new mode of cell death. Compared to apoptosis, autophagy, pyrolysis and necrosis, the biochemical regulation mechanism, morphological characteristics and genetic background of ferroptosis are also different from the classic cell death mode. It is caused by iron ion-dependent lipid peroxide damage. The morphological characteristics of ferroptosis cells under the electron microscope are mainly manifested as mitochondrial shrinkage, smaller volume, increased density of mitochondrial bilayer membrane, rupture of the outer membrane, mitochondrial cristae decrease or disappear, no obvious change in nucleus volume, and no chromatin concentration [28]. The biochemical characteristics are mainly manifested in the accumulation of iron ions and reactive oxygen species (ROS), cystine/glutamate antiporter inhibition, reduced glutathione synthesis, NAPDH oxidation, lipid peroxides accumulation on the cell membrane, etc. [29]. The mechanism of ferroptosis is the Fenton reaction between free iron ions and peroxides in the cell, so that the polyunsaturated fatty acids on the biofilm are further peroxidized, and its occurrence is regulated by a variety of genes. System Xc is a vital antioxidant system on the cell membrane. It is made up of two subunits coded by SLC7A11 as well as SLC3A2. Its main function is to take up cystine from the outside of the cell and discharge glutamate to the outside of the cell. After being taken up by System Xc, cystine is further reduced to cysteine via ethanethiol and other pathways to participate in the synthesis of glutathione. Glutathione then specifically and efficiently removes lipid peroxides with the participation of glutathione peroxidase 4 (GPX4), thereby inhibiting the occurrence of ferroptosis [29]. When SLC7A11 is inhibited, or the activity of GPX4 decreases, lipid peroxides cannot be metabolized through the glutathione reduction pathway. In turn, the Fenton reaction with ferrous ions produces a large amount of ROS, which causes lipid peroxides to accumulate on the cell membrane, thereby inducing ferroptosis. A number of studies have shown that inhibiting SLC7A11, GPX4 or other ferroptosis related molecules can reduce the production of glutathione, thereby promoting tumor cell ferroptosis and suppressing tumor growth and metastasis [30–33].

We quantitatively evaluated the effects of circKIF4A on ferroptosis of OS cells by total GSH/GSSG ratio and SLC7A11 protein expression, the results demonstrated that circKIF4A silencing significantly reduced SLC7A11 protein expression and GSH/GSSG ratio. circKIF4A is elevated in papillary thyroid carcinoma and the circKIF4A/miR-1231/GPX4 axis plays a crucial function in tumor proliferation as well as ferroptosis [25]. Similar to GPX4, SLC7A11 is also a molecule that is closely associated with ferroptosis and malignant progression of several tumors. The above findings imply that circKIF4A may play vital roles in the growth and metastasis of OS by circKIF4A/miR-1231/GPX4 axis, and this process may be closely related to ferroptosis. We will expand the sample size to further confirm the mechanism in the future.

## Conclusions

circKIF4A exerts if effects OS growth and metastasis via the circKIF4A-miR-515-5p-SLC7A11 axis. These findings provide a theoretical basis for the development of new therapeutic strategies and have potential prognostic implications for OS.

## Data Availability

All authors approved that all data and materials as well as software application or custom code support our published claims and comply with field standards. Data will be made available if needed.
